# Up to 15-Year Survival of Men and Women after Percutaneous Coronary Intervention Paid by the Brazilian Public Healthcare System in the State of Rio de Janeiro, 1999-2010

**DOI:** 10.5935/abc.20180184

**Published:** 2018-10

**Authors:** Christina Grüne de Souza e Silva, Carlos Henrique Klein, Paulo Henrique Godoy, Lucia Helena Alvares Salis, Nelson Albuquerque de Souza e Silva

**Affiliations:** 1 Instituto do Coração Edson Saad, Faculdade de Medicina, Hospital Universitário Clementino Fraga Filho, Universidade Federal do Rio de Janeiro, Rio de Janeiro, RJ - Brasil; 2 Escola Nacional de Saúde Pública Sergio Arouca - Fundação Oswaldo Cruz, Rio de Janeiro, RJ - Brasil; 3 Universidade Federal do Estado do Rio de Janeiro, Rio de Janeiro, RJ, Brasil

**Keywords:** Myocardial Revascularization, Coronary Artery Disease, Percutaneous Coronary Intervention, Mortality

## Abstract

**Background:**

Percutaneous coronary intervention (PCI) is the most frequently used invasive therapy for ischemic heart disease (IHD). Studies able to provide information about PCI's effectiveness should be conducted in a population of real-world patients.

**Objectives:**

To assess the survival rate of IHD patients treated with PCI in the state of Rio de Janeiro (RJ).

**Methods:**

Administrative (1999-2010) and death (1999-2014) databases of dwellers aged ≥ 20 years old in the state of RJ submitted to one single PCI paid by the Brazilian public healthcare system (*SUS*) between 1999 and 2010 were linked. Patients were grouped as follows: 20-49 years old, 50-69 years old and ≥ 70 years old, and PCI in primary PCI, with stent and without stent placement (bare metal stent). Survival probabilities in 30 days, one year and 15 years were estimated by using the Kaplan-Meier method. Cox hazards regression models were used to compare risks among sex, age groups and types of PCI. Test results with a p-value < 0.05 were deemed statistically significant.

**Results:**

Data of 19,263 patients (61 ± 11 years old, 63.6% men) were analyzed. Survival rates of men vs. women in 30 days, one year and 15 years were: 97.3% (97.0-97.6%) vs. 97.1% (96.6-97.4%), 93.6% (93.2-94.1%) vs. 93.4% (92.8-94.0%), and 55.7% (54.0-57.4%) vs. 58.1% (55.8-60.3%), respectively. The oldest age group was associated with lower survival rates in all periods. PCI with stent placement had higher survival rates than those without stent placement during a two-year follow-up. After that, both procedures had similar survival rates (HR 0.91, 95% CI 0.82-1.00).

**Conclusions:**

In a population of real-world patients, women had a higher survival rate than men within 15 years after PCI. Moreover, using a bare-metal stent failed to improve survival rates after a two-year follow-up compared to simple balloon angioplasty.

## Introduction

Ischemic heart disease (IHD) is the most frequent cause of death in adults^1^ and, although its age-standardized mortality rate has decreased over the last decades,^2^ IHD is still the cause of about 20% of all deaths worldwide.^2^^,^^3^

The most frequent invasive therapy for IHD is percutaneous coronary intervention (PCI).^4^ Since it was first performed,^5^^-^^7^ this procedure has been increasingly utilized, more expensive and possibly overused,^8^^,^^9^ although the majority of the studies conducted have evidenced just a few scenarios where PCI can be beneficial in IHD.^10^^,^^11^ Moreover, the information that guides physicians' decisions regarding its indication is mostly based on randomized controlled clinical trials (RCT), which usually enroll younger patients with fewer comorbid conditions than patients in the real-world, and exclude many treatment-related issues faced in clinical practice.^12^^,^^13^ Therefore, extrapolating PCI's effectiveness observed in RCTs to the real world-population may not be entirely appropriate.

This study aims at providing information about PCI's effectiveness in a real-world population by assessing short-, medium- and long-term survival rates of IHD patients treated with one single PCI, from 1999 to 2010, and paid by the Brazilian public healthcare system (*Sistema Único de Saúde* - *SUS*) in the state of Rio de Janeiro (RJ).

## Methods

### Study population and data collection

Data on PCI obtained at administrative databases of the state of RJ were analyzed retrospectively. The *DATASUS* administrative database of Authorization for Hospital Admission (*Autorização de Internação Hospitalar* - *AIH*) was consulted to gather data on PCI performed in public or private hospitals paid by the *SUS* between 1999 and 2010.

*SUS* is the Brazilian public healthcare system. It is funded from general government revenues, it is single, universal, hierarquical and integrated.^14^ *DATASUS* contains data of the Department of Healthcare Information of the Brazilian Ministry of Health, and it manages *SUS*' healthcare and financial information.^15^ *AIH* is a registry system^16^ for any admissions that occurs in any public or private hospital that maintain a convenant with the *SUS*.

Patient inclusion criteria: people who lived in the state of RJ, ≥ 20 years old, submitted to one single PCI between 1999 and 2010. Patient exclusion criteria: individuals submitted to coronary artery bypass grafting during the study period.

From the *AIH* database were obtained patients' name, date of birth, hospital admission and discharge, sex, address, mother´s name and type of PCI.

PCI procedures were classified according to the *AIH* database codes as described in a previous study^9^ as follows: a) PCI without stent placement (PCI-WS); b) PCI with stent placement (PCI-S); and c) primary PCI (PCI-P). During the study period the *SUS* would not pay for drug-eluting stents; therefore, PCI-S refers to the use of bare-metal stents.

The post-procedure outcome was death from any cause, and information on patients' death was obtained at the death database of the state of RJ from 1999 to 2014. In order to match information from both databases, *AIH* and deaths, Stata(r)14 probabilistic record linkage (Reclink) was used, once there is no common identification field between these two databases, and this essentially consists of a fuzzy merge. This method allows matching weights for each pre-defined variable, thus creating a new variable to hold the matching score in a zero-to-one scale, which indicates the probability that the pairs formed refer to the same patient. The pre-defined variables were patient´s name, date of birth and sex.

Pairs that scored = 1.00 (perfect matches) were considered the same patient. Pairs that scored ≥ 0.99 and < 1.00 were considered possible matches and were manually reviewed using mother´s name and address to define whether or not they were going to be considered the same patient. Pairs with lower scores were considered a "non-match".

In order to test the sensitivity and specificity of the probabilistic linkage method used, in-hospital deaths found at the *AIH* database were compared to the matching information from the death database. Out of a total of 357 in-hospital deaths found at the *AIH* database, 307 were found with the linkage process with the death database, and no false positives were detected. Therefore, the estimated sensitivity and specificity were 86% and 100%, respectively.

After the linkage process, patients were classified according to sex and the age groups 20-49, 50-69 and ≥ 70 years old. Underlying causes of death were obtained at the death database and classified according to the 10^th^ revision of the International Statistical Classification of Diseases and Related Health Problems (ICD-10)^17^ as IHD (codes I20 to I25) or non-IHD (any other code).

As the *AIH* database contains no information about the exact date of the PCI procedure, only the date of the patients` hospital admission and discharge, and as the average stay of these patients was 2 days,^9^ to analyze the survival rate the discharge date was considered day one. Short- and medium-term survival rates were defined as the probability of survival until day 30 and one year after discharge, respectively. As there are two possible discharge types at the *AIH* database - hospital discharge or death - short-term outcomes included in-hospital mortality rates. Long-term survival was defined as the probability of survival up to 10 or 15 years after hospital discharge for comparisons among types of PCI or between age groups and sex, respectively.

The study was approved by the ethics committee of *Hospital Universitário Clementino Fraga Filho* (*Faculdade de Medicina - UFRJ*) on 10/18/2012 (1148/12).

### Statistical analysis

Statistical analysis was performed based on data distribution. As the Shapiro-Wilk and Kolmogorov-Smirnov tests showed that age was not normally distributed, age distributions were described as median and interquartile ranges (P25-P75). Distribution of categorical variables was described as relative frequencies. The differences among groups were analyzed with the Kruskal-Wallis test for continuous variables or chi-square test for categorical variables. Probabilities of short-, medium- and long-term survival rates were estimated with the Kaplan-Meier survival method. Survival models were estimated with Cox proportional hazards regression to compare risks among age groups, sex and type of PCI; 95% confidence intervals (CI) were calculated to express the degree of uncertainty associated with the statistics for all analyses of subgroups. Stata 14^(r)^ was used for all analyses. Test results with a p-value < 0.05 were considered statistically significant.

## Results

Out of 22,735 patients, 3,472 were excluded and 19,263 were selected (63.6% men). Median (P25-P75) ages for men and women were 60 (52-68) and 62 (54-70) years, respectively (p < 0.05). The frequency distribution of the age groups 20-49, 50-69 and ≥70 years old for men and women was 16.2% and 13.1%, 63.9% and 60.1%, and 19.9% and 26.8%, respectively (p < 0.05).

**Figure 1:**
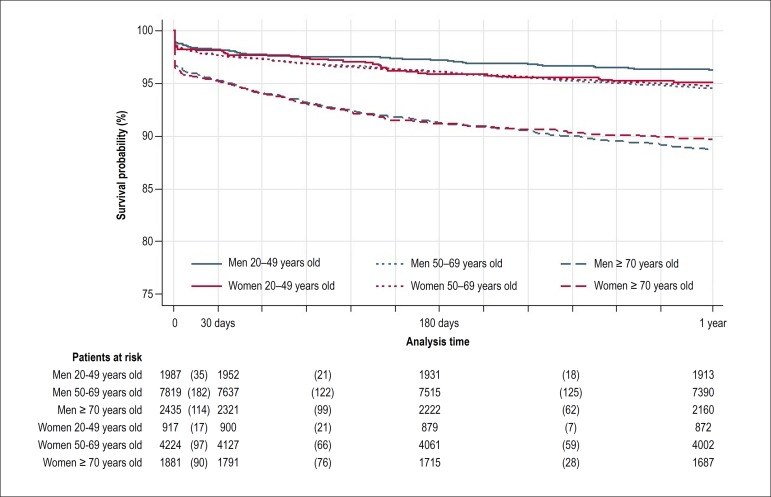
Kaplan-Meier survival estimates of patients submitted to one single percutaneous coronary intervention paid by SUS between 1999 and 2010 according to sex and age group in a one-year follow-up.

**Figure 2:**
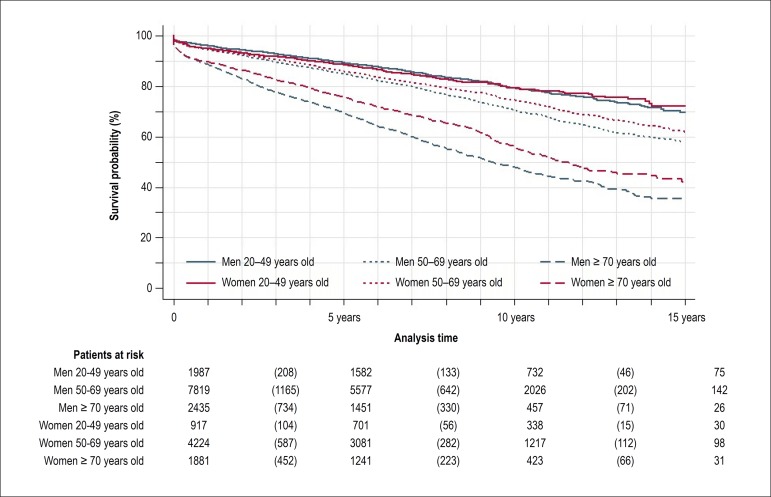
Kaplan-Meier survival estimates of patients submitted to one single percutaneous coronary intervention paid by SUS between 1999 and 2010 according to sex and age group until 15 years of follow-up.

Minimum and maximum follow-up were 4.0 and 15.0 years, respectively, and 5,433 patients (65.1% men) died during follow-up. Probabilities of survival and 95% CI for men and women were, respectively, short-term: 97.3% (97.0-97.6%) and 97.1% (96.6-97.4%), medium-term: 93.6% (93.2-94.1%) and 93.4% (92.8-94.0%), and long-term: 55.7% (54.0-57.4%) and 58.1% (55.8-60.3%). Men aged 20-49 years tended to have higher probability of survival in a 9-year follow-up, after which this tendency would reverse (Table 1). Men and women aged 50-69 years had the same probability of survival in a 180-day follow-up, after which women tended to have a higher probability of survival (Table 1). In the oldest age group men tended to have higher probability of survival, up to 180 days, after which that tendency would also reverse (Table 1). Figures 1 and 2 show Kaplan-Meier curves and estimates of survival according to sex and age group in one-year and 15-year follow-up, respectively. Table 2 shows Cox proportional hazards risks and 95% CI referring to age group and sex.

**Table 1:** Suvival proprabilities of patients submitted to a single percutaneous coronary intervention in the state of Rio de Janeiro paid by SUS between 1999-2010 according to age group and sex

Follow-up	20-49 years old		50-69 years old		≥70 years old	
	Men	Women	Men	Women	Men	Women
	(n = 1,987)	(n = 917)	(n = 7,819)	(n = 4,224)	(n = 2,435)	(n = 1,881)
	[% (95%Cl)]	[% (95%Cl)]	[% (95%Cl)]	[% (95%Cl)]	[% (95%Cl)]	[% (95%Cl)]
1 day	98.9 (98.3-99.3)	98.6 (97.6-99.2)	98.5 (98.2-98.8)	98.5 (98.1-98.9)	96.8 (96.0-97.4)	96.4 (95.4-97.1)
30 days	98.2 (97.5-98.7)	98.0 (96.9-98.8)	97.7 (97.3-98.0)	97.7 (97.2-98.1)	95.3 (94.4-96.1)	95.2 (94.1-96.0)
180 days	97.1 (96.3-97.8)	95.8 (94.2-96.9)	96.1 (95.7-96.5)	96.1 (95.5-96.6)	91.2 (90.0-92.3)	91.1 (89.7-92.3)
1 year	96.2 (95.3-97.0)	95.0 (93.4-96.2)	94.5 (94.0-95.0)	94.7 (94.0-95.4)	88.7 (87.3-89.9)	89.6 (88.2-90.9)
2 years	94.4 (93.3-95.3)	93.2 (91.4-94.7)	92.3 (91.6-92.8)	92.7 (91.9-93.5)	83.0 (81.5-84.4)	86.2 (84.6-87.7)
3 years	92.9 (91.7-94.0)	91.7 (89.7-93.3)	89.7 (89.0-90.3)	90.7 (89.8-91.6)	77.7 (76.0-79.3)	82.6 (80.8-84.3)
4 years	91.1 (89.8-92.3)	90.1 (88.0-91.8)	87.4 (86.6-88.1)	88.4 (87.4-89.4)	73.7 (71.9-75.4)	79.2 (77.3-80.9)
5 years	89.4 (87.9-90.7)	88.4 (86.2-90.3)	84.9 (84.0-85.6)	85.9 (84.8-86.9)	69.5 (67.7-71.3)	75.8 (73.8-77.7)
6 years	87.8 (86.2-89.2)	86.7 (84.2-88.8)	82.4 (81.5-83.2)	83.5 (82.3-84.6)	64.1 (62.1-66.0)	71.9 (69.8-74.0)
7 years	85.7 (84.0-87.2)	84.9 (82.3-87.1)	79.9 (79.0-80.9)	81.4 (80.2-82.6)	59.9 (57.8-62.0)	68.5 (66.2-70.7)
8 years	83.5 (81.6-85.1)	82.8 (79.9-85.2)	76.7 (75.6-77.7)	79.4 (78.0-80.7)	55.5 (53.2-57.6)	65.4 (63.0-67.7)
9 years	81.9 (80.0-83.7)	81.7 (78.7-84.2)	73.7 (72.5-74.8)	77.4 (76.0-78.8)	51.6 (49.3-53.9)	61.8 (59.3-64.3)
10 years	79.3 (77.1-81.3)	79.3 (76.1-82.1)	70.6 (69.3-71.8)	74.6 (73.0-76.1)	47.9 (45.5-50.3)	55.8 (53.0-58.5)
11 years	77.5 (75.2-79.6)	78.2 (74.9-81.2)	67.8 (66.4-69.1)	71.8 (70.0-73.5)	44.3 (41.8-46.8)	51.8 (48.9-54.7)
12 years	75.9 (73.4-78.1)	77.3 (73.9-80.4)	64.7 (63.1-66.1)	68.8 (66.9-70.7)	42.3 (39.6-44.9)	47.9 (44.7-51.0)
13 years	73.8 (71.1-76.3)	75.5 (71.7-78.9)	61.4 (59.7-63.1)	66.5 (64.3-68.6)	39.1 (39.6-42.0)	45.8 (42.4-49.0)
14 years	71.4 (68.2-74.4)	73.2 (68.6-77.3)	59.7 (57.8-61.6)	64.2 (61.7-66.6)	35.6 (32.3-39.0)	44.6 (41.1-48.0)
15 years	69.6 (65.8-73.1)	72.3 (67.3-76.7)	57.7 (55.4-60.0)	61.9 (58.9-64.9)	35.6 (32.3-39.0)	42.0 (37.5-46.4)

CI: confidence interval; SUS: Sistema Único de Saúde - Brazilian Public Healthcare System

**Table 2:** Cox proportional hazards risks and 95% confidence interval after short, medium and long-term follow-up in patients submitted to a single percutaneous coronary intervention in the state of Rio de Janeiro paid by SUS between 1999-2010 according age group, sex and type of procedure

	Short-term	Medium-term	Long-term
	HR (95% Cl)	HR (95% Cl)	HR (95% Cl)
**Age group**			
(50-69 years)/(20-49 years)	1.30 (0.97-1.75)	1.33 (1.09-1.61)	1.45 (1.32-1.58)
(≥70 years)/(20-49 years)	2.67 (1.97-3.62)	2.74 (2.24-3.35)	2.87 (2.61-3.16)
(≥70 years)/(50-69 years)	2.05 (1.71-2.46)	2.07 (1.84-2.33)	2.01 (1.89-2.13)
Sex*			
Women/Men - 20-49 years old	1.05 (0.59-1.88)	1.32 (0.91-1.92)	0.99 (0.83-1.19)
Women/Men - 50-69 years old	0.99 (0.78-1.27)	0.96 (0.81-1.13)	0.87 (0.81-0.94)
Women/Men - ≥70 years old	1.03 (0.79-1.36)	0.91 (0.76-1.10)	0.78 (0.71-0.86)
**Type of PCI^†^**			
(PCI-S)/(PCI-WS)	0.71 (0.59-0.85)	0.87 (0.77-0.98)	0.98 (0.92-1.04)
(PCI-P)/(PCI-WS)	3.34 (2.55-4.37)	2.32 (1.87-2.87)	1.32 (1.13-1.55)
(PCI-P)/(PCI-S)	4.72 (3.62-6.15)	2.68 (2.18-3.30)	1.38 (1.18-1.60)

Cl: confidence interval; PCI-P: primary percutaneous coronary intervention; PCI-S: percutaneous coronary intervention with stent placement; PCI-WS: percutaneous coronary intervention without stent placement; Medium-term: until 1 year of follow-up; Short-term: until 30 days of follow-up;

* Long-term: until 15 years of follow-up;

† Long-term: until 10 years of follow-up

**Figure 3:**
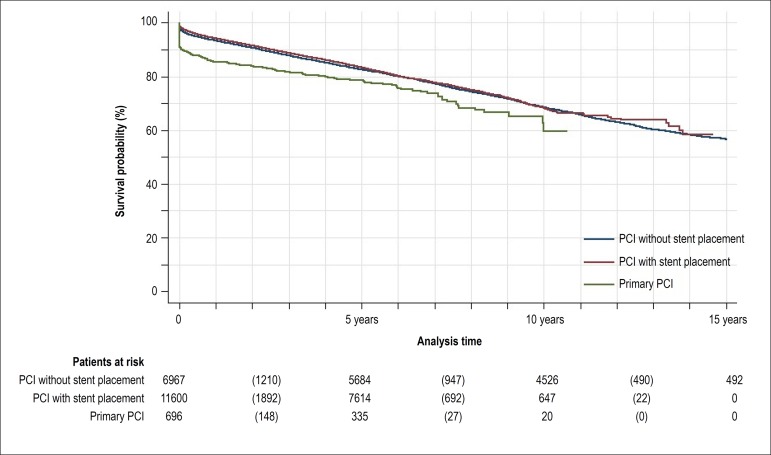
Kaplan-Meier estimates of survival for patients submitted to one single percutaneous coronary intervention (PCI) paid by SUS between 1999 and 2010 according to PCI type.

Concerning the type of PCI, patients who underwent PCI-P, PCI-WS and PCI-S were aged 61 ± 11, 60 ± 11, and 61 ± 10 years old, respectively (p < 0.05). A total of 175, 2,652 and 2,606 deaths occurred among patients submitted to PCI-P, PCI-WS, and PCI-S, respectively. Short-, medium- and long-term probabilities of survival for PCI-WS (n = 6,967) were 96.9% (96.5-97.3%), 93.4% (92.7-93.9%) and 68.6% (67.4-69.6%), respectively; for PCI-S (n = 11,600) were 97.8% (97.5-98.1%), 94.2% (93.7-94.6%) and 68.4% (67.0-69.7%), respectively; and for PCI-P (n = 696) were 89.8% (87.3-91.8%), 85.2% (82.3-87.6%) and 59.7% (49.8-68.2%), respectively. As PCI-S and PCI-P started to be paid by *SUS* in 2000 and 2004, respectively, long-term survival for the three procedures were measured in a 10-year follow-up for comparison purposes. Figure 3 shows Kaplan-Meier curves and estimates of survival and Table 2 presents Cox proportional hazards risks and 95% CI according to the type of PCI. In short- and medium-term follow-up, patients submitted to PCI-S had higher probability of survival than those submitted to PCI-WS, but after 2 years of follow-up their probabilities of survival became similar (HR 0.91, 95% CI 0.82-1.00, p = 0.062).

IHD was considered the underlying cause of death of 66.7%, 44.1% and 26.9% of the deaths that occured within 30 days, one year and 15 years after hospital discharge, respectively. During the entire follow-up period, PCI-P had the higher percentage of deaths due to IHD (49.1%) compared to PCI-WS (25.9%) and PCI-S (26.4%), p < 0.05.

## Discussion

This study has led to some important findings: 1) women tended to have slightly lower short- and medium-term probability of survival, but better long-term survival rates; 2) older patients had lower probabilities of survival; 3) differences in probability of survival changed slightly over time when PCI-P was compared to PCI with and without stent placement because the difference in the probability of survival was concentrated in the immediate period after the procedure; 4) although short- and medium-term survival rates were higher for patients submitted to PCI-S than for those submitted to PCI-WS, no difference was observed in the long-term survival rates between them; 5) the probabilities of survival observed were lower than those observed in RCTs.

Additionally, this study has some major strengths. First, it addressed a large number of patients (19,263) accompanied for more than 15 years, thus enabling the observation of important outcomes of interest in the future. Second, although only data from PCI paid by *SUS* were analyzed and, therefore, they could not mirror those observed with PCI paid exclusively with private resources, in the state of RJ the PCI paid by *SUS* accounts for the majority of the PCI procedures performed. Only about 25,3% and 33,5% of the population of the state of RJ in 2000 and 2010, respectively, had private health insurance,^18^ so at least 7 out of 10 of the PCI procedures performed in the state of RJ between 1999 and 2010 were certainly paid by *SUS*. Third, the data analyzed were from the third most populous Brazilian state and from 23 hospitals in the state of RJ, enabling the assessment of a broad range of patients and a high number of hospitals, which represent patients treated in a regular medical practice.

As to sex, former studies have examined the differences in survival or mortality rates between sex after a PCI. Although most agree that women present a higher prevalence of clinical risk factors and comorbidities when submitted to a PCI,^19^ there is conflicting evidence as to whether being a woman faces an independent risk of survival or mortality after a PCI. Data collected from German hospitals on PCI with or without stent placement in stable and acute coronary syndromes show that, after adjusting for age, women had higher in-hospital mortality rates than men only when the PCI was performed in the setting of ST-elevation myocardial infarction.^20^ In the CLARIFY study,^21^ similar rates of death for all causes after a one-year follow-up were observed for men and women with stable coronary artery disease submitted to PCI, after adjustment for baseline characteristics. On the other hand, data from the United Kingdom and Sweden^22^ showed that, when adjusting for age, being a woman was an independent predictor for all-cause mortality at 30 days and at one year after PCI performed for stable or acute coronary syndromes. In this study, even when clinical differences at baseline were not adjusted, women aged ≥50 years old tended to have lower survival rates than men the same age group in a 180-day follow-up, and in the youngest age group, women tended to have a lower survival probability even after over a 1-year follow-up.

As to long-term survival rates, most of the studies have shorter follow-up periods compared to those in this study. Berger et al.^23^ followed 4,284 patients in New York City for 3 years on average. Although men and women had the same in-hospital mortality rates, being a woman was independently associated with a reduction in hazards of long-term mortality. Similarly, the BARI study^24^ showed that when adjusting for baseline risk status, women had higher survival rates in a 5-year follow-up when treated with PCI for multivessel coronary artery disease. In the present study women also tended to have higher long-term survival rates, even though for the youngest age group this tendency only occurred after a 10-year follow-up.

The 2015 life table shows that in the general population in the state of RJ, women´s live expectancy is higher than men´s at the age groups addressed in this study: 22.6 and 18.8 years for women and men aged 60 years old, respectively, and 9.1 and 8.0 years for women and men aged ≥ 80 years old, respectively.^25^ However, it is not known if the survival of Brazilian men and women with coronary artery disease differ. In a study conducted in Norway with patients admitted to a hospital who had suffered a first episode of acute myocardial infarction, no age-adjusted sex-specific differences were observed in 28-day, one-year or 10-year case-fatality rate for patients aged <60 years.^26^ However, in patients aged ≥60 years, for the same periods, a lower case-fatality rate was evidenced in women. In Sweden, women that presented myocardial infarction, whether or not admitted to a hospital, over a 23-year period showed a 9% higher survival rate.^27^ Several attempts have been made in order to explain these conflicting results, such as biological attributes and social behaviors; however, those explanations are largely speculative. Regardless the causes, based on our results it seems that PCI reduces the gap in survival rates favoring women over men mainly among the cases involving younger patients (<50 years), and after some years following the intervention women have again a better probability of survival as observed in the general population.

As in other studies, here also older individuals had lower probabilities of survival than younger ones. The New York State Angioplasty Registry's data of patients submitted to emergency or elective PCI showed that when stratified by age group, overall in-hospital mortality rate in patients aged ≥ 80 years old was threefold higher than in patients aged 60-79 years, and sevenfold higher than in patients aged <60 years.^28^ A collaborative analysis from ten randomized trials,^29^ with a median follow-up of surviving patients of 5.9 years showed a 16% overall mortality rate of patients submitted to PCI done with balloon angioplasty or with bare-metal stents. As by age group, mortality rate in patients aged <55, 55-64 and ≥ 65 years old was 8%, 14% and 20%, respectively, showing a gradual effect of age in mortality.

Regarding the differences in outcomes after PCI with or without stent placement, while there is no doubt that bare-metal stent placement reduces the rate of restenosis and revascularization,^30^ most RCTs have failed to show any advantage as to mortality rates of bare-metal stent placement over simple balloon angioplasty. The BENESTENT group has found no differences in in-hospital mortality and mortality rates at 7 months, one year and 5 years, in patients with stable angina submitted to PCI-S or simple balloon angioplasty.^31^^,^^32^ A meta-analysis of RCTs comparing both procedures in the setting of non-acute coronary artery disease have shown just a small benefit in overall mortality rates with the routine use of stent, corresponding to an average of three, five and six additional lives saved per 1,000 patients treated at 30 days, 6 months and 12 months, respectively.^33^ However, it was not possible to guarantee that this small additional benefit related to mortality rates was due to stent placement instead of to unbalanced co-interventions once more aggressive post-intervention therapy was observed in the stent group. As for acute myocardial infarction, Suryapranata et al.^34^ showed that in a follow-up of 24 months the rates of reinfarction and of subsequent target-vessel revascularization were higher in patients submitted to simple balloon angioplasty, but no difference was observed in mortality rates between the stent group and the balloon group.

As for observational studies, the analysis of the New York State´s Coronary Angioplasty Reporting System data^35^ showed that in-hospital mortality rates were not different between PCI with and without stent placement, but the gap between the mortality rates in the two procedures widened about six months after the procedure, favoring PCI-S, and after that the gap remained constant for a two-year follow-up. Our study also observed a higher survival rate for patients submitted to PCI-S; however, the survival rate gap between the two procedures was larger at the beginning of the follow-up, getting narrower in longer follow-up periods and, finally, from 2 to 10 years no more differences in the survival rates were observed. Therefore, after these results, future studies should be conducted to address whether PCI using drug-eluting stents shows different results when compared to bare-metal stent or simple balloon angioplasty, and whether stent placement is cost-effective against simple balloon angioplasty for the public healthcare system in the state of RJ.

Finally, the death rates observed in this study are higher than those in RCTs. In a RCT conducted in the United States and in Canada with patients with stable or unstable coronary artery disease,^36^ 0.4% and 1.2% of the patients submitted to PCI-S and simple balloon angioplasty died, respectively, compared to 4.3% and 5.2%, respectively, in our study at 6 months of follow-up. Boden et al.^37^ showed a 7.6% cumulative death rate in 4.6 years of follow-up in patients with stable coronary artery disease submitted to PCI, (~3% with drug-eluting stent), while in our study 16.3% of the patients submitted to PCI-S died until 5 years of follow-up. In a continued follow-up of 53% of the original population from the former study, Sedlis et al.^38^ showed that 25% of the patients submitted to PCI died within 15 years against 28.2% of deaths observed in this study. These discrepancies are likely to be explained by the problematic extrapolation of RCTs' findings to the general population because of their restrictive inclusion and exclusion criteria. Therefore, this observational study is more likely to provide an indication of what is being achieved in the daily medical practice with a population of patients assisted by the Brazilian public healthcare system and, thus, observational studies should be deemed complementary to RCTs' results. So, indications of PCI, especially in cases of stable IHD and in older patients, have to be questioned once the survival rates observed in such cases were lower than those expected when just clinical treatment has been used. We have to stress that the cases selected were submitted to one single procedure during the study period and they probably represent cases of better prognosis in the large spectrum of clinical presentations of IHD.

Some limitations inherent to observational studies should be highlighted. The data provided were limited to those included in the *AIH* database. The *AIH* database was created for administrative purposes and hence it does not include some important clinical information such as comorbidities, medications prescribed, number of vessels affected and patients' socioeconomic status, which might have influenced our results. Furthermore, these secondary databases did not follow strict data collection protocols and may be considered of lower quality in comparison to the data collected in RCTs. Yet, today the *AIH* database is the best tool available in Brazilian´s public healthcare system for this type of study due to its comprehensiveness and accessibility.

## Conclusion

This study reports the probability of survival in 30 days, one year and 15 years of follow-up of a large number of patients submitted to one single PCI procedure paid by the Brazilian public healthcare system in the state of Rio de Janeiro. Women were prone to have a slightly lower survival probability than men in 30-day and one-year follow-up, but women had a higher survival probability within 15 years, especially when they were older. Additionally, patients submitted to PCI procedures without stent placement had a lower probability of survival within 30 days and one year, although no difference was observed after a two-year follow-up regarding the use of stents. These findings, which mirror the medical practice performed in a real-world population may help physicians make decisions regarding indicating the PCI considering the questions raised about the true benefits of this procedure.
